# A Recombinant Trivalent Fusion Protein F1–LcrV–HSP70(II) Augments Humoral and Cellular Immune Responses and Imparts Full Protection against *Yersinia pestis*

**DOI:** 10.3389/fmicb.2016.01053

**Published:** 2016-07-05

**Authors:** Shailendra K. Verma, Lalit Batra, Urmil Tuteja

**Affiliations:** Microbiology Division, Defence Research & Development Establishment, GwaliorIndia

**Keywords:** plague, *Yersinia pestis*, HSP70(II), cytokine, F1–LcrV, F1–LcrV–HSP70(II)

## Abstract

Plague is one of the most dangerous infections in humans caused by *Yersinia pestis*, a Gram-negative bacterium. Despite of an overwhelming research success, no ideal vaccine against plague is available yet. It is well established that F1/LcrV based vaccine requires a strong cellular immune response for complete protection against plague. In our earlier study, we demonstrated that HSP70(II) of *Mycobacterium tuberculosis* modulates the humoral and cellular immunity of F1/LcrV vaccine candidates individually as well as in combinations in a mouse model. Here, we made two recombinant constructs *caf1–lcrV* and *caf1–lcrV–hsp70(II)*. The *caf1* and *lcrV* genes of *Y. pestis* and *hsp70* domain II of *M. tuberculosis* were amplified by polymerase chain reaction. Both the recombinant constructs *caf1–lcrV* and *caf1–lcrV–hsp70(II)* were cloned in pET28a vector and expressed in *Escherichia coli.* The recombinant fusion proteins F1–LcrV and F1–LcrV–HSP70(II) were purified using Ni-NTA columns and formulated with alum to evaluate the humoral and cell mediated immune responses in mice. The protective efficacies of F1–LcrV and F1–LcrV–HSP70(II) were determined following challenge of immunized mice with 100 LD_50_ of *Y. pestis* through intraperitoneal route. Significant differences were noticed in the titers of IgG and it’s isotypes, i.e., IgG1, IgG2b, and IgG3 in anti- F1–LcrV–HSP70(II) sera in comparison to anti-F1–LcrV sera. Similarly, significant differences were also noticed in the expression levels of IL-2, IFN-γ and TNF-α in splenocytes of F1–LcrV–HSP(II) immunized mice in comparison to F1–LcrV. Both F1–LcrV and F1–LcrV–HSP70(II) provided 100% protection. Our research findings suggest that F1–LcrV fused with HSP70 domain II of *M. tuberculosis* significantly enhanced the humoral and cellular immune responses in mouse model.

## Introduction

*Yersinia pestis* (*Y. pestis*), etiologic agent of plague is accountable for several outbreaks throughout the world and caused the deaths of millions of people right through the history. In spite of substantial advancement in its prevention and cure, ~50,000 human cases of plague have been reported during the last two decades (WHO, 2005). Worldwide, an average of 4000 human plague cases are reported every year, with outbreaks erupting from time to time which cause mass casualties (Green et al., 2014). Therefore, now, World Health Organization (WHO) considered plague as a re-emerging disease (Schrag and Wiener, 1995; WHO, 2005).

Humans are very prone to plague infection and the etiology of the infection is reliant to the route and source of the infection, consequential in one of the three main clinical forms of plague: bubonic, septicaemic, and pneumonic (Williamson, 2009). Transmission of *Y. pestis* bacilli to a human begins with an accidental bite of an infected flea and causing the bubonic plague. *Y. pestis* occasionally reaches to the lungs or inhalation of aerosolized *Y. pestis* follow-on pneumonic plague. This pneumonic form of plague has the highest fatality rate with a 1–3 days incubation period. This pneumopathy is 100% lethal usually less than 3 days if no treatment is provided (Butler, 2013).

The possible use of aerosolized *Y. pestis* as a bio-weapon agent is also a serious risk due to its high fatality rate and transmission of human-to-human capability. Centers for Disease Control (CDC) has classified to *Y. pestis* in a category of biosafety level-3 (BSL-3) organisms. There are antibiotics available for the treatment of plague but recently antibiotic resistant plague bacilli have been reported (Galimand et al., 1997; Guiyoule et al., 2001). It is documented that antibiotic resistant strains of *Y. pestis* can be prepared in laboratory by transferring the plasmids containing antibiotic resistance genes (Guiyoule et al., 2001; Chain et al., 2004). Therefore, antibiotics are likely to be less effective in the future against intentional/natural outbreaks of plague. In such a scenario, vaccines may be one of the only left over options to control the fatality toll in humans. Therefore, it is imperative to develop an effective and safe plague vaccine.

Currently, there is no licensed vaccine available against plague. Earlier, the strain EV76 of *Y. pestis* (live attenuated) was used and confers protection in humans (Girard, 1963; Feodorova and Motin, 2011). However, *Y. pestis* has been considered a major obstacle in its use as live attenuated or killed vaccine due to its genetic instability and several risk factors (side effects) associated with it (Chain et al., 2004; Cui et al., 2014). Recently, many recombinant protein based vaccines candidates have been developed; amongst them only two vaccines, i.e., RypVax^TM^ and rF1V^TM^ are the most superior in clinical trials. These vaccines mainly rely on a combination of two proteins, F1 and LcrV (Williamson et al., 1995; Heath et al., 1998). These two antigens F1 and LcrV are the main and effective targets which provide protective immunity against plague (Burrows, 1956; Girard, 1963). Recombinant vaccine candidates are generally formulated with aluminum hydroxide gel, a human compatible adjuvant and thus are strong inducers of humoral immune response but poor inducers of cellular immunity (Mannhalter et al., 1985; Dinc et al., 2014). The cellular immune response, however, plays an important role to develop protective immunity against plague (Smiley, 2008). Despite of adequate humoral immune response, F1–V-based vaccines provide poor and inconsistence protection in African Green monkeys, probably due to a weak cellular immune response.

In our earlier studies (Batra et al., 2014), we have shown that F1 and LcrV in formulation with HSP70(II) of *M. tuberculosis* induced an improved cellular immune response in mouse model. HSP70(II) modulated the cell mediated immunity as there was a significant difference in the expression of IFN-γ and TNF-α in mice immunized with a cocktail of F1, LcrV, and HSP70(II) in comparison to mice immunized with F1+LcrV. In continuation to our efforts to develop a more effective vaccine against plague, here we made two recombinant constructs *caf1–lcrV* and *caf1–lcrV*–*hsp70(II)* successfully expressed in *Escherichia coli* and purified the recombinants proteins by metal affinity chromatography upto homogeneity. To evaluate the protective potential and immune responses, Balb/C mice were immunized with F1–LcrV and F1–LcrV–HSP70(II). There was a significant difference in IgG response in the sera of immunized mice with F1–LcrV–HSP70(II) in comparison to F1–LcrV. The F1–LcrV–HSP70(II) induced significantly elevated levels of IL-2, IFN-γ, TNF-α in comparison to F1–LcrV vaccine candidate. Immunized mice were experimentally infected with 100 LD_50_ of *Y. pestis* (S1 strain) via intra-peritoneal route.

## Materials and Methods

### Ethics Statement

All the protocols for conducted experiments using mice approved by Institutional Animal Ethics Committee (IAEC) of Defence Research and Development Establishment *via* registration number 37/Go/C/1999/CPCSEA. All the procedures of good laboratory animal care were followed during the course of experiment. The experimental mice were maintained according to the recommendations of committee for the purpose of control and supervision of experiments on animals (CPCSEA), Govt. of India.

### Bacterial Strains, Plasmids, and Reagents

*Escherichia coli* host strain BL21 (DE3) and DH5α were purchased from Invitrogen, USA. *Y. pestis* (S1 strain), an Indian clinical isolate (Verma et al., 2013; Batra et al., 2014) was collected from DRDE repository (DB182YEPE1) used for challenge experiments. The expression vector pET28a+ was from Novagen, USA. Live *Y. pestis* cultures and challenge experiments were completed in high containment facility (BSL-3), at DRDE, Gwalior.

### Cloning of *caf1–lcrV* and *caf1–lcrV–hsp70(II)* Recombinant Constructs in pET Vector

*Yersinia pestis* cultures were grown on Brain Heart Infusion (BHI) agar plate and incubated at 28°C for 48 h. Genomic DNA was extracted by DNeasy Blood and Tissue kit (Qiagen, Germany) according to the manufacturer’s instruction. The genes *caf1* and *lcrV* of *Y. pestis* were amplified by polymerase chain reaction (PCR). The gene *hsp70(II)* of *M. tuberculosis* was amplified by PCR from an earlier cloned plasmid (Batra et al., 2014). The descriptions of used primers are given in **Table 1**. To prepare the *caf1*–*lcrV* construct, first *caf1* was ligated with pET28a vector using *Nco I* and *Bam HI* and later *lcrV* ligated in the same plasmid using *BamHI* and *Sal I* restriction enzymes. Similarly, in order to prepare the *caf1*–*lcrV–hsp70(II)* construct, *lcrV* reverse primer (R) was replaced with reverse primer (R′) that carrying the *Sac I* restriction site. First *caf1* was ligated with pET28a vector using the same restriction enzymes as mentioned above followed by *lcrV* using *Bam HI* and *Sac I* followed by *hsp70(II)* using *Sac I* and *Hind III* restriction enzymes. These two ligated constructs were transformed in DH5α cells and positive clones were selected using kanamycin (50 μg/ml).

**Table 1:** List of oligos.

Gene	Oligos	Restriction sites	Size (bp)
*caf1*	F-5′-ataccatgggcATGAAAAAAATCAGTTCCGTTATCG-3′	*Nco I*	513
	R-5′-taggatccTTGGTTAGATACGGTTACGGTTACAG-3′	*Bam HI*	
lcrV	F-5′-taggatccATTAGAGCCTACGAACAAAACCCACA-3′	*Bam HI*	981
	R-5′-tagtcgacTTTACCAGACGTGTCATCTAGCAGACG-3′	*Sal I*	
	R′-5′-tagagctcTTTACCAGACGTGTCATCTAGCAGACG-3′	*Sac I*	
hsp70(II)	F-5′-tagagctcGAGAAGGAGCAGCGAATCCTG-3′	*Sac I*	630
	R-5′-taaagcttCGGGGTAACATCAAGCAGCAG-3′	*Hind III*	

### Expression and Purification of F1–LcrV and F1–LcrV–HSP70(II)

The individual recombinant construct corresponding to *caf1–lcrV* and *caf1–lcrV–hsp70(II)* cloned in pET28a vector was transformed in BL21(DE3) cells. The positive colonies were selected on Luria-Bertani (LB) agar plates using kanamycin (50 μg/ml). The single colony of each construct, i.e., *caf1–lcrV* and *caf1–lcrV–hsp70(II)* was inoculated individually into 5 ml of LB broth and grown at 37°C for overnight. Next day, 500 ml of LB broth was inoculated with 1% of overnight grown culture and incubated at 37°C. When the optical density (OD_600_) reached upto ~0.6, the cultures were induced with 1 mM isopropylthiogalactoside (IPTG) and grown for 4 h at 37°C. The cultures were pelleted by centrifugation at 10,000 × *g* for 10 min at 4°C. The cells were lysed in sample buffer and analyzed by SDS–PAGE for the expression. The recombinant fusion proteins F1–LcrV and F1–LcrV–HSP70(II) were purified using 8 M urea by metal affinity chromatography using Ni-NTA columns (Qiagen, USA). The purified antigens were run on 10% SDS–PAGE and anti-histidine tag antibodies (Qiagen, USA) recognized both recombinant proteins in Western blot experiments. The purified proteins were subjected under dialysis. The dialyzed proteins were concentrated using Amicon ultra centrifugal filter devices (Millipore). Protein concentrations were measured by BCA method using BCA kit (Sigma, USA).

### Immunization of Mice

In order to test the protective efficacy and immune responses of F1–LcrV and F1–LcrV–HSP70(II) vaccine candidates against *Y. pestis*, female Balb/C mice (6 week old) were collected from DRDE animal facility, Gwalior. The animals were divided in two batches; each batch was containing three groups (8 mice/group), i.e., Control group; F1–LcrV group; and F1–LcrV–HSP70(II) group (**Figure 3A**). Batch-I was utilized for the study of humoral immune response (IgG antibody) and challenge experiments against *Y. pestis*. Batch-II was utilized for the study of cell mediated immune response (cytokine profiling). Animals were immunized subcutaneously with 20 μg/mouse of each purified protein in formulation with aluminum hydroxide gel. The animals of control group received PBS only. The immunizations were given on day 0; day 14; and day 21. All the immunized animals were subjected for blood collection on day 0, 21, and 28 as shown in **Figure 3B**. Sera were separated from the blood samples for the study of humoral immune response and IgG isotyping.

### IgG Antibody Response

The end point titers of IgG antibodies were measured by enzyme-linked immunosorbent assay (ELISA) in the sera of immunized and control group animals. In brief, 96 well ELISA plates were coated individually with F1–LcrV and F1–LcrV–HSP70(II) fusion proteins using 100 ng/well each antigen in 0.05 M carbonate buffer, pH 9.6. The coated plates were incubated for overnight at 4°C. After three extensive washings with 0.05% Tween 20 in PBS (PBS-T), the plates were blocked with 3% bovine serum albumin (BSA), incubated for 2 h at 37°C. Plates were washed, test sera collected from immunized and control groups after first and second boosters were serially diluted in triplicate wells (100 μl/well). The plates were incubated for 1 h at 37°C. After five extensive washings, plates were probed with anti-mouse horseradish peroxidase (HRP) labeled IgG (Sigma, USA) raised in rabbit at 1:20,000 dilutions in PBS, incubated for 1 h at 37°C. The plates were washed as earlier and the reaction was developed with *o*-phenylenediamine dihydrochloride as substrate and after 10 min stopped by 2N H_2_SO_4_. The optical density (OD) was measured at 490 nm by a multimode reader (Biotek, USA).

### IgG Isotyping

In order to characterize the IgG isotypes, microtiter plates were coated with F1–LcrV and F1–LcrV–HSP70(II) (0.1 μg/well) and blocked with BSA as mentioned above. IgG isotypes sera from animal groups *viz*; control, F1–LcrV and F1–LcrV–HSP70(II) collected after second booster, added at 1:1000 dilution in PBS in triplicate wells. Plates were incubated for 1 h at 37°C, washed and again incubated with goat anti-mouse isotype specific antibodies IgG1, IgG2a, IgG2b, and IgG3 (Sigma, USA) at 1:1000 dilutions for 1 h at 37°C. Detection of bound isotype specific antibodies was estimated with rabbit anti-goat IgG HRP conjugated (1:5000 dilutions). The reaction was stopped with 2N H_2_SO_4_ and optical density (OD) was measured at 490 nm.

### Cellular Immune Response

Animal groups of batch-II were sacrificed and their spleens were removed in aseptic conditions. To measure the cytokines single cell suspension of spleen cells was prepared. The cells (1 × 10^6^ cells/well) of each group were seeded in triplicates in a culture plate. The cells were stimulated with F1–LcrV or F1–LcrV–HSP70(II) antigens or ConA (5 μg/ml each). After 48 h, the supernatants of cultured spleen cells were collected. The induced level of IL-2; IL-4; IL-10; IFN-γ; and TNF-α were estimated by ELISA kit (BD Biosciences, USA) according to the manufacturer’s instructions. The concentrations of cytokines in picograms per milliliter (pg/ml) were measured with the help of standard curves using recombinant cytokines as standard with the ELISA kit.

### Challenge of Animals

*Yersinia pestis* (S1 strain) from the frozen stocks were streaked on BHI agar plate and incubated at 28°C for 48 h. Single colony from BHI agar plate was further inoculated in 5 ml of BHI broth and grown at 28°C for 48 h. The LD_50_ of *Y. pestis* (S1 strain, an Indian clinical isolate) has been determined earlier (Verma et al., 2013; Batra et al., 2014). To determine the protective potential of F1–LcrV and F1–LcrV–HSP70(II) vaccine candidates, animals of batch-I were infected with 100 LD_50_ of *Y. pestis* via intraperitoneal route after 1 month of last booster. Infected animals were observed for their survival for 30 days of challenge (**Figure 3A**).

### Statistical Analysis

Results were presented as the means value ± standard deviation (SD). Statistical comparisons among various immunized groups were analyzed by SigmaStat 3.5, one way ANOVA, All Pairwise Multiple Comparison Procedure (Fisher LSD Method). *P < 0.05; **P < 0.01; ***P < 0.001; **^#^***P* < 0.001. Survival curves were prepared by Kaplan–Meier method using GraphPad Prism 6.0 demo version (****P < 0.0001).

## Results

### Cloning of *caf1–lcrV* and *caf1–lcrV–hsp70(II)* in pET Vector

**FIGURE 1:**
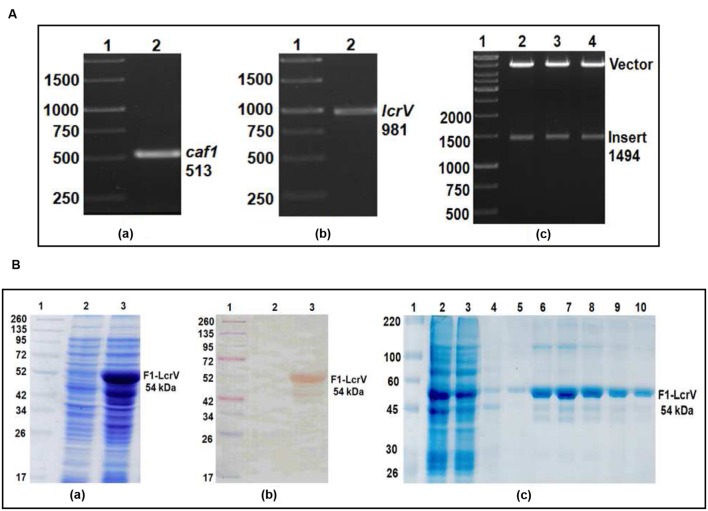
**(A)** PCR amplification of *caf1* and *lcrV* genes of *Yersinia pestis* & cloning of *caf1–lcrV* in pET vector. *caf1* gene (a); Lane 1, 1Kb DNA ladder; Lane 2, *caf1* gene product of 513 bp. *lcrV* gene (b); Lane 1, 1Kb DNA ladder; Lane 2, *lcrV* gene product of 981 bp. Screening of positive clones of *caf1–lcrV* fusion product after ligation in pET28a vector (c); Lane 1, 1 Kb DNA ladder; Lane 2–4, released inserts (*caf1–lcrV*) of ~1500 bp after restriction digestion from pET vector. **(B)** Recombinant F1–LcrV fusion protein expression profile and purification. (a) SDS–PAGE analysis of F1–LcrV protein expression. Lane 1, Pre-stained protein marker; Lane 2, Uninduced *Escherichia coli* cell lysate clone; Lane 3, Induced *E. coli* cell lysate (b) Western blot analysis of F1–LcrV protein showing reaction with anti HIS antibody. Lane 1, Pre-stained protein marker; Lane 2, Uninduced *E. coli* cell lysate clone; Lane 3, Induced *E. coli* cell lysate. (c) Purification of F1–LcrV protein. Lane 1, Protein marker; Lane 2, Cell lysate; Lane 3, Flow through; Lane 4, Wash; Lane 5–10, Eluted fractions of protein showing F1–LcrV protein of 54 kDa.

**FIGURE 2:**
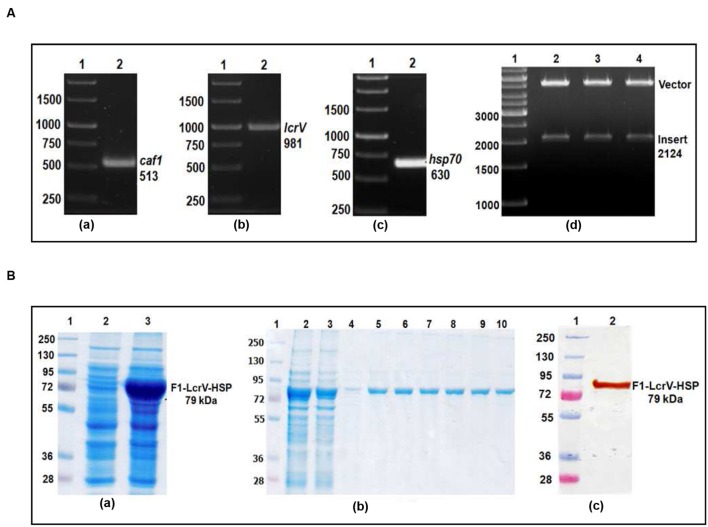
**(A)** PCR amplification of *caf1*, *lcrV* genes of *Y. pestis* and hsp70(II) of *Mycobacterium tuberculosis* & cloning of *caf1–lcrV–hsp70(II)* in pET vector. *caf1* gene (a); Lane 1, 1 Kb DNA ladder; Lane 2, *caf1* gene product of 513 bp: *lcrV* gene (b); Lane 1, 1 Kb DNA ladder; Lane 2, *lcrV* gene product of 981 bp: *hsp70(II)* gene (c); Lane 1, 1 Kb DNA ladder; Lane 2, *hsp70(II)* gene product of 630 bp. Screening of positive clones of *caf1–lcrV–hsp70(II)* fusion product after ligation in pET28a vector (d); Lane 1, 1 Kb DNA ladder; Lane 2–4, released inserts (*caf1–lcrV*) of 2124 bp after restriction digestion from pET vector. **(B)** Recombinant F1–LcrV-HSP70(II) trivalent fusion protein expression profile & purification. (a) SDS–PAGE analysis of F1–LcrV–HSP70(II) protein expression: Lane 1, Protein marker; Lane 2, Uninduced *E. coli* cell lysate clone; Lane 3, Induced *E. coli* cell lysate. (b) Purification of F1–LcrV–HSP70(II) protein: Lane 1, Protein markers; Lane 2, Cell lysate; Lane 3, Flow through; Lane 4, Wash; Lane 5–10, Eluted fractions of purified protein showing F1–LcrV–HSP70(II) fusion protein of 79 kDa. (c) Western blot analysis of purified F1–LcrV–HSP70(II) protein showing reaction with anti HIS antibody: Lane 1, Pre-stained protein marker; Lane 2, purified F1–LcrV–HSP70(II) protein.

In order to prepare F1–LcrV and F1–LcrV–HSP70(II) proteins, *caf1* of 513 bp and *lcrV* 981 bp of *Y. pestis* and hsp70(II) of *M. tuberculosis* were amplified by PCR (**Figures 1** and **2**) with the help of primers manipulated with compatible restriction site (**Table 1**). First, *caf1* and *lcrV* were linked with help of *Bam HI* restriction enzymes (**Figure 1A(c)**). The *caf1–lcrV* construct was carrying *NcoI* and *Sal I* restriction sites at 3′ and 5′, respectively. Similarly, to prepare the *caf1–lcrV–hsp70(II)*, *caf1, lcrV*, and *hsp70(II)* were linked with help of *Bam HI* and *Sac I* restriction sites (**Figure 2A(d)**). The *caf1–lcrV–hsp70(II)* construct was carrying *Nco I* and *Hind III* restriction sites at 3′ and 5′, respectively. The DNA constructs, *caf1–lcrV* of ~1500 bp encoding F1–LcrV a bivalent protein (~54 kDa) and *caf1–lcrV–hsp70(II)* of ~2130 bp encoding F1–LcrV–HSP70(II) a trivalent protein (~79 kDa) were cloned in pET28a+ vector using *Nco I/Sal I* and *Nco I/Hind III* restriction sites. The individual pET vector carrying the *caf1–lcrV* and *caf1–lcrV–hsp70(II)* was transformed in DH5α cells and the positive clones were selected on LB agar plates with kanamycin. The in-frames of the cloned constructs were validated by nucleotide sequencing (Chromous Biotech, India).

### Expression and Purification of F1–LcrV and F1–LcrV–HSP70(II)

One of the positive clones corresponding to *caf1–lcrV* and *caf1–lcrV–hsp70(II)* was individually transformed in *E. coli* host BL-21 (DE3). For the expression of F1–LcrV and F1–LcrV–HSP70(II), 500 ml LB broth was inoculated to each clone and the cultures were induced with IPTG. The expressed proteins F1–LcrV and F1–LcrV–HSP70(II) were analyzed by SDS–PAGE. The proteins SDS–PAGE profiles of un-induced and IPTG-induced cultures for F1–LcrV and F1–LcrV–HSP70(II) are shown in **Figures 1B(a)** and **2B(a)**, respectively. In Western blot experiments, anti-histidine antibody recognized F1–LcrV and F1–LcrV–HSP70(II) protein bands corresponding to their molecular weights as shown in **Figures 1B(b)** and **2B(c)**. In order to purify the fusion proteins, i.e., F1–LcrV and F1–LcrV–HSP70(II), the recombinant constructs were engineered to bear the 6X-Histine tag at C-terminus. Both the cultures were lysed using native conditions and both the proteins were found present in pellet fractions. The purification was carried out under denaturing conditions by solubilizing the pellet of each culture separately in 8 M urea. Both the proteins were purified by Ni-NTA affinity chromatography using earlier published protocol (Verma et al., 2013). The purified F1–LcrV and F1–LcrV–HSP70(II) proteins were analyzed by SDS–PAGE as shown in **Figures 1B(c)** and **2B(b)**, respectively. Both purified proteins were concentrated and the obtained yield of F1–LcrV and F1–LcrV–HSP70(II) was 2.0 and 2.5 mg/L of shake flask cultures, respectively.

### IgG Antibody Response

**FIGURE 3:**
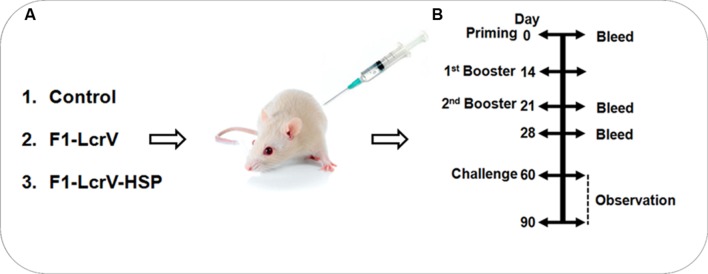
**Representation of animal groups and the schedule of vaccination activities. (A)** Prepared groups of Balb/C mice (8/group) for vaccination studies with F1–LcrV and F1–LcrV–HSP70(II) in formulation with alum. **(B)** Schematic representation of vaccination schedule, blood collection for humoral and cell mediated studies and challenge experiments.

**FIGURE 4:**
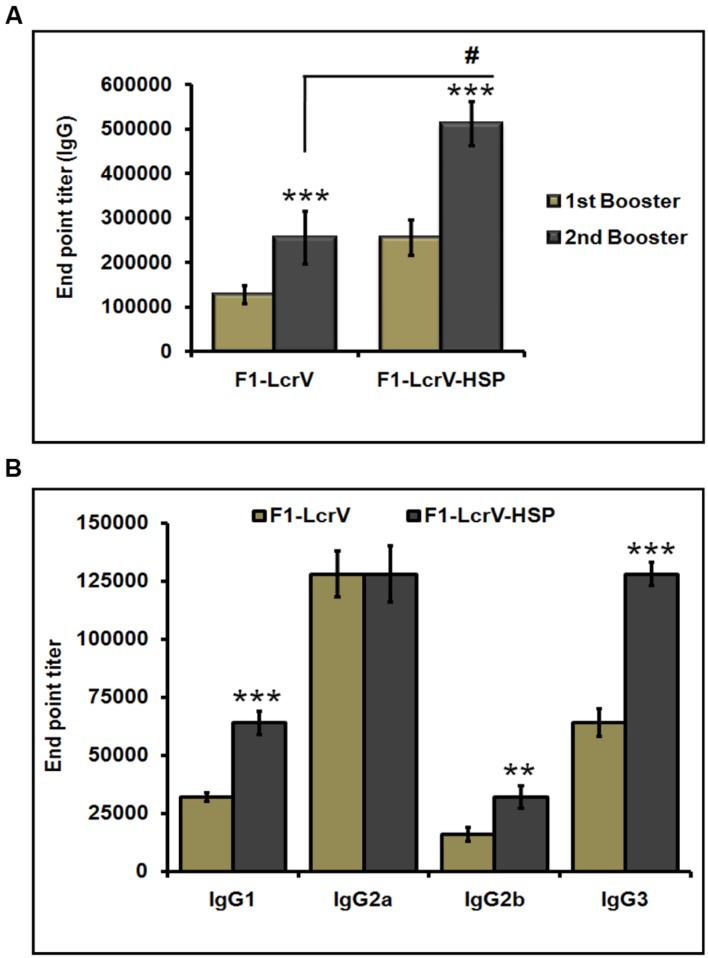
**End point titers of IgG antibody and IgG isotypes in the sera of immunized mice. (A)** IgG end point titers were estimated by ELISA. Serum samples were collected after first and second boosters from vaccinated animal groups, i.e., F1–LcrV and F1–LcrV–HSP70(II)). **(B)** The IgG isotypes; IgG1, IgG2a, IgG2b, and IgG3 were measured by ELISA. End point titers of IgG isotypes in sera samples collected after second booster from vaccinated mice with F1–LcrV and F1–LcrV–HSP70(II) antigens. Analysis was done by one way ANOVA, all Pairwise Multiple Comparison Procedure (Fisher LSD Method). ** *P* < 0.01; *** *P* < 0.001; **^#^***P* < 0.001.

To measure the IgG endpoint titers, sera were collected 7 days after first and second injections, respectively, from all the immunized animals as shown **Figure 3B**. The cut-off value for the assays was calculated as the mean OD (+2 SD) from sera of control group assayed at 1:200 dilutions. The IgG endpoint titers were calculated as reciprocal of the highest serum dilution giving an OD more than the cut-off. The IgG endpoint titer to F1–LcrV was observed 1.28 × 10^5^ and 2.56 × 10^5^ in the sera of F1–LcrV group after first and second booster, respectively. The IgG endpoint titer to F1–LcrV–HSP70(II) was observed 2.56 × 10^5^ and 5.12 × 10^5^ in the sera of F1–LcrV–HSP70(II) group after first and second booster, respectively. A significant difference (**^#^***P* < 0.001) was observed in the endpoint titer of F1–LcrV–HSP70(II) immunized sera in comparison to F1–LcrV immunized sera (**Figure 4A**).

### IgG Isotypes

The endpoint titers of IgG isotypes were also determined similarly as described above. IgG isotypes were measured by ELISA specific to F1–LcrV and F1–LcrV–HSP70(II) in sera samples collected after 7 days of second boosters from all the immunized groups. Significantly elevated levels of IgG1, IgG2b and IgG3 were observed to F1–LcrV–HSP70(II) (**Figure 4B**) in comparison to F1–LcrV group whereas; there was no difference in the expression of IgG2b to both the groups.

### Cellular Immune Response

**FIGURE 5:**
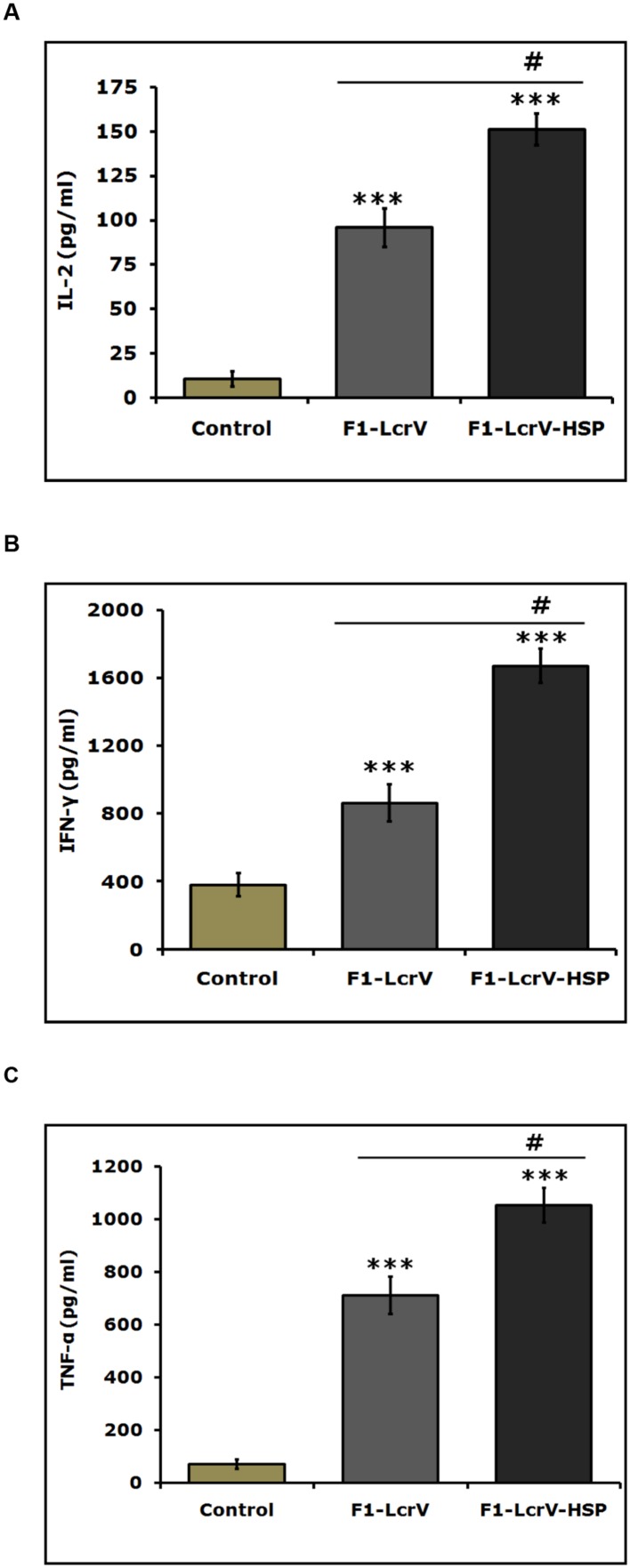
**Estimation of cytokines in vaccinated animal groups.** Splenocytes isolated from immunized BALB/c mice with F1–LcrV and F1–LcrV–HSP70(II) including control group. Splenocytes were stimulated with F1–LcrV and F1–LcrV–HSP70(II) antigens (5 μg/ml each) and after 48 h, the expression levels of cytokines were measured. The expression levels of cytokines **(A)** IL-2, **(B)** IFN-γ, and **(C)** TNF-α were calculated in picograms per milliliter (pg/ml) as shown in graphs. Data represent the average of 8 mice/group ± SD of the results determined. One way ANOVA was applied for analysis. *** *P* < 0.001; **^#^***P* < 0.001.

The expression levels of cytokines, i.e., IL-2, IL-4, IL-10, IFN-γ, and TNF-α of all immunized animal groups were measured in supernatants of splenocytes stimulated with required antigen/s. A significance difference (***P < 0.001) was noticed in the expression levels of IL-2 (**Figure 5A**), IFN-γ (**Figure 5B**), and TNF-α (**Figure 5C**) in F1–LcrV and F1–LcrV–HSP70(II) immunized animal groups in comparison to control group. There was no significant difference in the expression levels of IL-4 and IL-10 (data not shown). The splenocytes from all animal groups were induced with ConA and responded non-specifically. We also observed a significant difference (**^#^***P* < 0.001) in the expression of IL-2, IFN-γ and TNF-α in F1–LcrV–HSP70(II) immunized group in comparison to F1–LcrV group.

### Protection Studies

**FIGURE 6:**
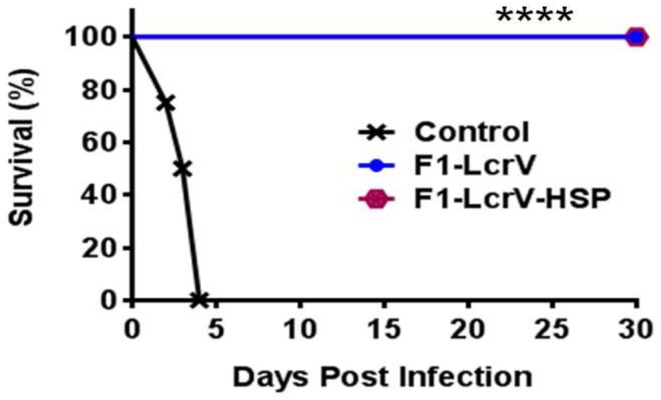
**Vaccination with F1–LcrV and F1–LcrV–HSP70(II) protects mice against *Y. pestis* (S1 strain).** The vaccinated and control group animals were experimentally infected via *i.p.* route with 100 LD_50_ of virulent *Y. pestis.* The survivals of animals were monitored for 30 days after challenge. The survival curve of both the vaccine candidates F1–LcrV and F1–LcrV–HSP70(II) was determined by Kaplan Meier’s method to calculate percentage survivals (**** *P* < 0.0001).

To study the protective efficacy, the immunized animals were challenged with 100 LD_50_ of *Y. pestis* (S1 strain) including control group. Survivals of the animals were monitored for 30 days post challenge (**Figure 6**). Both vaccine candidates F1–LcrV and F1–LcrV–HSP70(II) provided 100% protection from the *Y. pestis* challenged mice (****P < 0.0001). There was no protection observed in control groups.

## Discussion

Plague is a historic and lethal infectious disease which is so far prevalent in many regions of the recent world and caused by *Y. pestis*, a potential bio-warfare agent. After the attacks of anthrax in 2001 in USA, it became preferential to stockpile the safe and ideal vaccines against anthrax and plague to protect a mass population against these deadly dangerous bioterror attacks. Nevertheless, there is no plague vaccine has been approved worldwide so far. The possible explanations comprise inconsistence protection, weak stability, poor immunogenicity and the problems in formulation strategies. Modern antigens designing strategies and new vaccine proposals will be helpful to conquer these problems. These new strategies would be of immense attention to develop an effective plague vaccine. In this study, we generated a new trivalent fusion protein that provided full protection against plague in a mouse model.

The F1, a capsular protein and LcrV, a type III secretion protein are the surface-exposed antigens of *Y. pestis* and have been the leading antigens to develop a subunit vaccine against plague for many years (Anderson et al., 1998; Heath et al., 1998; Williamson, 2009). The vaccine based on these two candidates (F1/LcrV) is poorly immunogenic and mainly induces humoral immune response. Despite of generating high titers of anti-F1/LcrV specific IgG antibodies, some vaccinated animals succumbed to challenge (Bashaw et al., 2007). The protection conferred by live attenuated *Salmonella* expressing LcrV antigen in mouse model did not show any correlation with LcrV antibody titers (Garmory et al., 2003). Similarly, vaccination of non-human primates specially, African Green monkeys with recombinant F1–LcrV fusion protein impart partial and inconsistent protection against plague and the level of protection did not correlate with the generated humoral immune response either F1 or LcrV antibody titers (Bashaw et al., 2007; Rosenzweig et al., 2011; Williamson and Oyston, 2013). These conclusions, strongly advocate that antibodies titers alone cannot sufficiently envisage the efficacy of F1/LcrV based vaccines.

In order to improve the efficacy of F1/LcrV-based vaccines, several approaches were undertaken like genetic modification of the antigens, e.g., by mutating cysteine amino acid in rF1/V (Goodin et al., 2007) or by deleting the immunosuppressive region of LcrV (DeBord et al., 2006), use of alternate adjuvants like alum, poly-L-lactide microspheres, Monophosphoryl lipids, TiterMax, and flagellin as an molecular adjuvant (Honko et al., 2006; Jones et al., 2006; Feodorova and Motin, 2012), delivery platforms (Garmory et al., 2003; Palin et al., 2007; Yang et al., 2007) and formulations with additional antigen/s that might play the role of an immunomodulator/s or an immunoregulator/s to enhance the immune response.

Heat shock proteins (HSPs) are best known to modulate the immune response (Hauser and Chen, 2003; Pockley, 2003; Robert, 2003; Binder, 2006) and stimulating effective T-cell responses to model antigens (Rico et al., 1998) and pathogen-derived antigens as well (Chen et al., 2000; Huang et al., 2000). Recently, domain II of heat shock protein, HSP70(II) of *M. tuberculosis* has been proven for inducing the T-cell response by many scientists (Massa et al., 2005; Dhakal et al., 2013). Recombinant fusion constructs Ovalbumin-HSP70(II) elicit CD8^+^ cytotoxic T lymphocytes specific to ovalbumin (Huang et al., 2000). Suzue and Young (1996) proved that HSP70(II) of *M. tuberculosis* augment the humoral and cellular immune response to the p24 protein of HIV-1. Subsequently, Wang et al. (2002), reported that the C-terminal portion (amino acids 359–610) of the mHSP70 mainly elicits Th-1 type of cytokines (IL-2, TNF-α, and IFN-γ) and higher serum IgG2a and IgG3 isotypes of antibodies.

In our earlier studies (Batra et al., 2014), we have characterized heat shock protein 70 domain II, HSP70(II) of *M. tuberculosis* as an immunomodulator to augment the immune response of F1 and LcrV vaccine candidates. It has been proven in our earlier studies that HSP70(II) not only enhances the protective efficacy of F1 and LcrV but also modulates the cellular immune responses and significantly increases the expression levels of IL-2, IFN-γ, and TNF-α in mouse model. HSP70(II) significantly increased the IFN-γ secreting CD4^+^ and CD8^+^ T cells in the mice immunized with a cocktail of F1, LcrV, and HSP70(II) comparison to mice immunized with F1and LcrV. In the present study to reduce the labor, cost, and time, for generation of individual protein, we made two fusion constructs, F1–LcrV and F1–LcrV–HSP70(II) and evaluated in mice. We were able to demonstrate that fusion construct of F1–LcrV–HSP70(II) could provide a significant increase in the production of IgG antibody in comparison to F1–LcrV. A significant difference was also noticed in the titers of IgG isotypes, i.e., IgG1, IgG2b, and IgG3 in F1–LcrV–HSP70(II) vaccinated group in comparison to F1–LcrV. We also noticed the significant difference in the expression of IL-2, IFN-γ and TNF-α by the splenocytes in F1–LcrV–HSP70(II) immunized animal group in comparison to F1–LcrV. Both recombinant fusion proteins F1–LcrV and F1–LcrV–HSP70(II) provided full protection against virulent *Y. pestis* in a mouse model. The results generated with these two fusion proteins were in agreement with our earlier findings (Batra et al., 2014).

## Conclusion

The study is worthwhile as the fusion of HSP70(II) with F1–LcrV significantly enhanced the humoral (IgG and its isotypes; IgG1, IgG2b, and IgG3) and cellular (IL-2, IFN-γ, and TNF-α) immune responses. These research findings open up new avenues for vaccine development against plague, however, the findings further need to be evaluated in non-human primates.

## Author Contributions

SV and UT were involved in designing the experiments, data analysis and manuscript preparation. Experiments performed by SV, UT, and LB.

## Conflict of Interest Statement

The authors declare that the research was conducted in the absence of any commercial or financial relationships that could be construed as a potential conflict of interest.
